# Supplementary material for: Neurotype matching in monogamous rodents is modulated by early-life sleep experience
Source: bioRxiv. 2025 Sep 26:2025.09.24.678442. Preprint. [Version 2] doi: 10.1101/2025.09.24.678442 (PMC12485720; doi:10.1101/2025.09.24.678442)
Supplement: Supplement 1 [file media-1.pdf]

figure panel	dyad type or sex	effect	value	direction to divider	distance to divider	locomotion speed
2A	all dyads	sex	deg fr	1, 27	1, 27	1, 27
			F; P	**=37.78; <0.001**	=0.13; =0.722	=4.03; =0.055
		time	deg fr	71, 1917	71, 1917	71, 1917
			F; P	=7.92; <0.001	=9.06; <0.001	=19.10; <0.001
		interaction	deg fr	71, 1917	71, 1917	71, 1917
			F; P	**=3.13; <0.001**	=1.01; =0.460	**=1.73; <0.001**
2B	matched dyads	sex	deg fr	1, 14	1, 14	1, 14
			F; P	=7.72; =0.015	=1.50; =0.241	=0.85; =0.373
		time	deg fr	71, 994	71, 994	71, 994
			F; P	=4.46; <0.001	=3.51; <0.001	=11.10; <0.001
		interaction	deg fr	71, 994	71, 994	71, 994
			F; P	=1.06; =0.340	=0.73; =0.955	=0.76; =0.929
	mixed dyads	sex	deg fr	1, 12	1, 12	1, 12
			F; P	**=72.53; <0.001**	=1.30; =0.276	=4.52; =0.055
		time	deg fr	71, 852	71, 852	71, 852
			F; P	=4.33; <0.001	=6.06; <0.001	=8.88; <0.001
		interaction	deg fr	71, 852	71, 852	71, 852
			F; P	**=4.52; <0.001**	=1.05; =0.373	**=1.63; =0.001**
2C	Ctrl-Ctrl	sex	deg fr	1, 6	1, 6	1, 6
			F; P	=3.04; =0.132	=0.39; =0.553	=0.15; =0.715
		time	deg fr	71, 426	71, 426	71, 426
			F; P	=3.54; <0.001	=2.80; <0.001	=5.74; <0.001
		interaction	deg fr	71, 426	71, 426	71, 426
			F; P	=0.77; =0.915	=0.49; =1.000	=0.41; =1.000
	ELSD-ELSD	sex	deg fr	1, 7	1, 7	1, 7
			F; P	=4.27; =0.078	=1.17; =0.315	=1.71; =0.232
		time	deg fr	71, 497	71, 497	71, 497
			F; P	=2.14; <0.001	=1.51; =0.007	=5.39; <0.001
		interaction	deg fr	71, 497	71, 497	71, 497
			F; P	=0.89; =0.720	=0.61; =0.995	=1.00; =0.485
	Ctrl-ELSD	sex	deg fr	1, 6	1, 6	1, 6
			F; P	**=35.70; <0.001**	=5.02; =0.066	=1.10; =0.335
		time	deg fr	71, 426	71, 426	71, 426
			F; P	=3.86; <0.001	=3.78; <0.001	=3.13; <0.001
		interaction	deg fr	71, 426	71, 426	71, 426
			F; P	**=2.31; <0.001**	**=2.57; <0.001**	=0.41; =1.000
	ELSD-Ctrl	sex	deg fr	1, 5	1, 5	1, 5
			F; P	**=37.71; =0.002**	=0.05; =0.838	=3.62; =0.115
		time	deg fr	71, 355	71, 355	71, 355
			F; P	=1.24; =0.111	=2.77; <0.001	=7.65; <0.001
		interaction	deg fr	71, 355	71, 355	71, 355
			F; P	**=2.52; <0.001**	=0.35; =1.000	**=3.46; <0.001**
2D	males	dyad type	deg fr	1, 12	1, 12	1, 12
			F; P	=8.26; =0.014	=0.96; =0.346	=0.03; =0.865
		time	deg fr	71, 852	71, 852	71, 852
			F; P	=3.15; <0.001	=3.44; <0.001	=8.36; <0.001
		interaction	deg fr	71, 852	71, 852	71, 852
			F; P	**=1.58; =0.002**	=0.54; =0.999	=0.55; =0.999
	females	dyad type	deg fr	1, 12	1, 12	1, 12
			F; P	=1.42; =0.257	=2.05; =0.178	=0.28; =0.607
		time	deg fr	71, 852	71, 852	71, 852
			F; P	=7.47; <0.001	=7.12; <0.001	=9.06; <0.001
		interaction	deg fr	71, 852	71, 852	71, 852
			F; P	=1.09; =0.302	=0.97; =0.540	=1.30; =0.055

Table 1. Statistics for Figure 2. Degrees of freedom, F, and P values were obtained using two-way ANOVA with time bins as repeated measures. P values < 0.01 were highlighted with bold font, except for repeated measure effects.
