# Supplementary material for: Neurotype matching in monogamous rodents is modulated by early-life sleep experience
Source: bioRxiv. 2025 Sep 26:2025.09.24.678442. Preprint. [Version 2] doi: 10.1101/2025.09.24.678442 (PMC12485720; doi:10.1101/2025.09.24.678442)
Supplement: Supplement 2 [file media-2.pdf]

figure panel	dyad type or sex	effect	value	chasing	attacking	pointing	behaving alone	huddling
3A	all dyads	sex	deg fr	1, 27	1, 27	1, 27	1, 27	1, 27
			F; P	=4.79; =0.038	**=16.28; <0.001**	=1.11; =0.302	=0.00; =1.000	=0.00; =1.000
		time	deg fr	79, 2133	79, 2133	79, 2133	79, 2133	79, 2133
			F; P	=19.20; <0.001	=21.83; <0.001	=8.95; <0.001	=8.64; <0.001	=13.89; <0.001
		interaction	deg fr	79, 2133	79, 2133	79, 2133	79, 2133	79, 2133
			F; P	**=3.89; <0.001**	**=11.30; <0.001**	=0.95; =0.607	=0.00; =1.000	=0.00; =1.000
3B	matched dyads	sex	deg fr	1, 14	1, 14	1, 14	1, 14	1, 14
			F; P	=0.31; =0.588	=3.77; =0.073	=0.72; =0.409	=0.00; =1.000	=0.00; =1.000
		time	deg fr	79, 1106	79, 1106	79, 1106	79, 1106	79, 1106
			F; P	=8.72; <0.001	=12.96; <0.001	=4.54; <0.001	=6.06; <0.001	=8.66; <0.001
		interaction	deg fr	79, 1106	79, 1106	79, 1106	79, 1106	79, 1106
			F; P	=0.61; =0.997	**=4.25; <0.001**	=0.64; =0.993	=0.00; =1.000	=0.00; =1.000
	mixed dyads	sex	deg fr	1, 12	1, 12	1, 12	1, 12	1, 12
			F; P	=6.87; =0.022	**=15.96; =0.002**	=0.35; =0.565	=0.00; =1.000	=0.00; =1.000
		time	deg fr	79, 948	79, 948	79, 948	79, 948	79, 948
			F; P	=12.11; <0.001	=13.83; <0.001	=4.77; <0.001	=3.08; <0.001	=5.38; <0.001
		interaction	deg fr	79, 948	79, 948	79, 948	79, 948	79, 948
			F; P	**=5.25; <0.001**	**=8.37; <0.001**	=0.88; =0.757	=0.00; =1.000	=0.00; =1.000
3C	Ctrl-Ctrl	sex	deg fr	1, 6	1, 6	1, 6	1, 6	1, 6
			F; P	=0.06; =0.822	=8.63; =0.026	=0.35; =0.578	=0.00; =1.000	=0.00; =1.000
		time	deg fr	79, 474	79, 474	79, 474	79, 474	79, 474
			F; P	=4.49; <0.001	=3.82; <0.001	=2.33; <0.001	=1.42; =0.015	=2.75; <0.001
		interaction	deg fr	79, 474	79, 474	79, 474	79, 474	79, 474
			F; P	=0.52; =1.000	**=3.58; <0.001**	=0.29; =1.000	=0.00; =1.000	=0.00; =1.000
	ELSD-ELSD	sex	deg fr	1, 7	1, 7	1, 7	1, 7	1, 7
			F; P	=0.91; =0.373	=0.31; =0.598	=3.47; =0.105	=0.00; =1.000	=0.00; =1.000
		time	deg fr	79, 553	79, 553	79, 553	79, 553	79, 553
			F; P	=6.16; <0.001	=9.99; <0.001	=2.48; <0.001	=6.15; <0.001	=6.72; <0.001
		interaction	deg fr	79, 553	79, 553	79, 553	79, 553	79, 553
			F; P	=0.68; =0.981	**=1.45; =0.010**	**=1.76; <0.001**	=0.00; =1.000	=0.00; =1.000
	Ctrl-ELSD	sex	deg fr	1, 6	1, 6	1, 6	1, 6	1, 6
			F; P	=3.54; =0.109	=9.11; =0.023	=0.67; =0.444	=0.00; =1.000	=0.00; =1.000
		time	deg fr	79, 474	79, 474	79, 474	79, 474	79, 474
			F; P	=6.24; <0.001	=6.58; <0.001	=3.84; <0.001	=2.30; <0.001	=3.53; <0.001
		interaction	deg fr	79, 474	79, 474	79, 474	79, 474	79, 474
			F; P	**=3.86; <0.001**	**=6.76; <0.001**	=0.65; =0.990	=0.00; =1.000	=0.00; =1.000
	ELSD-Ctrl	sex	deg fr	1, 5	1, 5	1, 5	1, 5	1, 5
			F; P	=2.92; =0.148	=5.97; =0.058	=0.06; =0.816	=0.00; =1.000	=0.00; =1.000
		time	deg fr	79, 395	79, 395	79, 395	79, 395	79, 395
			F; P	=5.91; <0.001	=7.17; <0.001	=1.96; <0.001	=1.91; <0.001	=2.85; <0.001
		interaction	deg fr	79, 395	79, 395	79, 395	79, 395	79, 395
			F; P	**=2.20; <0.001**	**=3.28; <0.001**	=0.57; =0.999	=0.00; =1.000	=0.00; =1.000
3D	males	dyad type	deg fr	1, 12	1, 12	1, 12	1, 12	1, 12
			F; P	=0.60; =0.454	=0.01; =0.943	=1.72; =0.215	=0.67; =0.430	=0.11; =0.746
		time	deg fr	79, 948	79, 948	79, 948	79, 948	79, 948
			F; P	=11.40; <0.001	=6.25; <0.001	=5.16; <0.001	=6.94; <0.001	=11.35; <0.001
		interaction	deg fr	79, 948	79, 948	79, 948	79, 948	79, 948
			F; P	=0.90; =0.716	=0.82; =0.873	=0.66; =0.990	=0.45; =1.000	=0.35; =1.000
	females	dyad type	deg fr	1, 12	1, 12	1, 12	1, 12	1, 12
			F; P	**=10.81; =0.006**	=5.82; =0.033	=2.77; =0.122	=0.67; =0.430	=0.11; =0.746
		time	deg fr	79, 948	79, 948	79, 948	79, 948	79, 948
			F; P	=13.05; <0.001	=19.21; <0.001	=8.67; <0.001	=6.935; <0.001	=11.35; <0.001
		interaction	deg fr	79, 948	79, 948	79, 948	79, 948	79, 948
			F; P	**=2.70; <0.001**	**=4.42; <0.001**	=0.36; =1.000	=0.448; =1.000	0.35; =1.000

Table 2. Statistics for Figure 3. Degrees of freedom, F, and P values were obtained using two-way ANOVA with time bins as repeated measures. P values < 0.01 were highlighted with bold font, except for repeated measure effects.
