# Supplementary material for: Neurotype matching in monogamous rodents is modulated by early-life sleep experience
Source: bioRxiv. 2025 Sep 26:2025.09.24.678442. Preprint. [Version 2] doi: 10.1101/2025.09.24.678442 (PMC12485720; doi:10.1101/2025.09.24.678442)
Supplement: Supplement 3 [file media-3.pdf]

figure panel	sex direction	effect	value	
5B	male-to-female	dyad type	deg fr	1, 12
			F; P	=0.02; =0.904
		behavior pair	deg fr	11, 132
			F; P	=123.86; <0.001
		interaction	deg fr	11, 132
			F; P	=0.69; =0.747
	female-to-male	dyad type	deg fr	1, 12
			F; P	=0.01; =0.955
		behavior pair	deg fr	11, 132
			F; P	=138.55; <0.001
		interaction	deg fr	11, 132
			F; P	**=5.23; <0.001**
5D	male-to-female	dyad type	deg fr	1, 12
			F; P	=0.80; =0.389
		behavior pair	deg fr	19, 228
			F; P	=70.03; <0.001
		interaction	deg fr	19, 228
			F; P	=0.87; =0.618
	female-to-male	dyad type	deg fr	1, 12
			F; P	=5.13; =0.043
		behavior pair	deg fr	19, 228
			F; P	=68.26; <0.001
		interaction	deg fr	19, 228
			F; P	**=2.64; <0.001**

Table 3. Statistics for Figure 5. Degrees of freedom, F, and P values were obtained using two-way ANOVA with behavior pairs as repeated measures. P values < 0.01 were highlighted with bold font, except for repeated measure effects.
